# Mutual effects of stratification and mixed convection on Williamson fluid flow under stagnation region towards an inclined cylindrical surface

**DOI:** 10.1016/j.mex.2017.10.007

**Published:** 2017-10-24

**Authors:** Khalil Ur Rehman, Abid Ali Khan, M.Y. Malik, Usman Ali

**Affiliations:** Department of Mathematics, Quaid-i-Azam University, Islamabad, 44000, Pakistan

**Keywords:** Williamson fluid model, Temperature stratification, Concentration stratification, An inclined cylinder, Mixed convection, Shooting method

## Abstract

We have found that few attempts are reported on Williamson fluid flow yields by an inclined cylindrical surface. To be more specific, Williamson fluid flow regime characteristics under stagnation point region especially when it is manifested with mutual interaction of double stratification and mixed convection effects are not proposed as yet. Therefore, we have considered stagnation point mixed convection Williamson fluid flow brought by an inclined cylinder in the presence of temperature and concentration stratification phenomena. Further, the fluid flow is entertained through no slip condition i-e the velocity of particles is directly related to velocity of cylindrical surface due to stretching. The physical situation within the real concerned constraints is translated in terms of differential equations as a boundary value problem. To make implementation of computational algorithm possible, following steps are taken into account

• The PDE’s are transformed into ODE’s by using suitable transformation.
• The resulting boundary value problem is converted into an initial value problem.
• These constructed ordinary differential equations are solved computationally by shooting technique charted with Runge-Kutta scheme.
• The effect logs of an involved physical flow parameters are explored by way of graphical outcomes and tabular values.

## Graphical abstract

**Figure:**
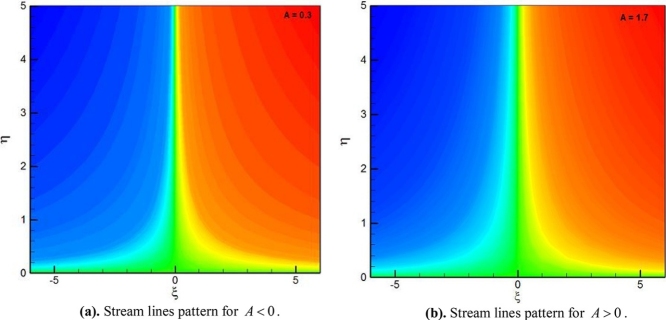

## Method details

The study of non-Newtonian fluids flow brought by stretching surfaces is always remains a topic of great interest and has received outstanding attention by the researchers due to their numerous applications in an industrial and engineering process. To be more specific, pulps, sugar solutions, tomato ketchup, and shampoos are the well-known examples of non-Newtonian fluids. In non-Newtonian fluids, there exists non-linear bond between deformation rate and shear stresses so, it is difficult to narrate the accurate salient features of non-Newtonian fluid models. To clip out the complete description of non-Newtonian fluids flow different model are proposed by scientists like Maxwell fluid model (1867), Barus fluid model (1893), Bingham Herschel-Bulkly (1922), Ostwald-de Waele power law model (1923), Williamson fluid model (1929), Eyring fluid model (1936), Burgers fluid model (1939), Generalized Burgers (1939), Reiner-Rivlin fluid model (1945), Oldroyd-A (1950), Oldroyd-B fluid model (1950), Oldroyd-8 constants (1950), Glen fluid model (1955), Rivlin-Ericksen (1955), Criminale-Ericksen-Filbey (1957), Sisko fluid model (1958), Kaye-Bernstein-Kearsley-Zapas (K-BKZ, 1963), Seely fluid model (1964), Cross fluid model (1965), FENE-P (1966), Carreau fluid model (1972), Carreau-Yasuda fluid model (1972), Johnson-Segalman fluid model (1977), Johnson-Tevaarwerk (1977), Phan-Thien-Tanner fluid model (1978), Giesekus fluid model (1982), FENE-CR (1988), Ellis fluid model, Blatter fluid model (1995), and White-Metzner, Rolie-Poly. In short, the interest of researchers and scientists immensely increased towards rheological features of non-Newtonian fluids like Abel et al. [1] discussed non-Newtonian fluid flow characteristics in the presence of magnetic field, heat and mass transfer effects. Tan and Masuoka [2] identified Stokes first problem by an incorporating second grade fluid by means of heated boundary. The mutual properties of heat and mass subject to viscoelastic fluid model was taken by Sanjayanand and Khan [3]. Ishak et al. [4] presented variable heat flux characteristics for micropolar fluid model. Whereas, the impact of magnetic field on power law fluid flow an induced by stretching flat surface was studied by Chen [5]. The combined properties of heat and mass transfer regarding peristaltic movement for a Jeffrey fluid was discussed by Nadeem and Akbar [6]. Ashorynejad et al. [7] probed nanofluid flow yields by stretching cylindrical surface in the presence of heat transfer phenomena along with magnetic field effect. The influence of thermal radiation on MHD nanofluid flow with heat transfer was given by Sheikholeslami et al. [8]. Recently, the non-Newtonian fluid flow with heat and mass transfer manifested with bio-convection was taken by Raju et al. [9]. More recently, Salahuddin et al. [10] deliberated non-Newtonian fluid flow characteristics by means of stretching cylindrical surface.

To be more specific, out of these non-Newtonian fluid models, Williamson model is quoted as fluid with pseudo-plastic features. In addition, Williamson fluid model admits shear thinning properties. The industrial and biological liquids that obey the Williamson fluid are polymers, melts/solution, ketchup blood, paint, and whipped cream to mention just a few. Williamson [11] exposed this model to express pseudo-plastic physiognomies with both characteristics of minimum and maximum viscosity effects. Then rest of motivated researchers explore more heads for this non-Newtonian fluid category like Aksoy et al. [12] derived first boundary layer equations subject to Williamson fluid model and offered symmetry analysis. Akbar et al. [13] found numerical solution of Williamson nanofluid flow by using both fourth and fifth order RK–Fehlberg method in an irregular channel. Nadeem et al. [14] examined Williamson fluid flow over a stretched surface using homotopy analysis method. Nadeem and Hussain [15] found series solution of Williamson nanofluid flow over a stretching sheet. Hayat et al. [16] also studied Williamson fluid flow analytically using homotopy analysis method in attendance of viscous dissipation, Newtonian Joule heating. Malik and Salahuddin [17] discussed numerical simulation of MHD Williamson fluid flow near stagnation point using RK–Fehlberg method over a stretched cylinder. Malik et al. [18] observed flow of Williamson fluid numerically with heat generation/absorption and variable thermal conductivity effects over a stretched cylinder using RK–Fehlberg method. Hayat et al. [19] highlighted Soret and Dufour impacts in Williamson fluid flow under the influence of thermal radiation and viscous dissipation over an unsteady stretched surface Salahuddin et al. [20] explored numerical solution of MHD Williamson fluid flow with Cattaneo–Christov heat flux and variable thickness effects past a stretched surface. The thermal stratification effects on Williamson fluid flow towards a stretching cylinder was considered by Bilal el at. [21].

The assessed literature survey reflects that an investigation subject to Williamson fluid flow brought by an inclined stretching cylindrical surface manifested with double stratification is not reported as yet. Therefore, the key aim of present attempt is to inspect the influence of double stratification effects along with mixed convection on Williamson fluid flow under stagnation point region carried by an inclined stretching cylinder. To the best of our knowledge it seems to be a first attempt in this direction to discuss the double stratification effects on Williamson fluid flow along an inclined stretching cylinder under stagnation point region in the presence of mixed convection effects. For this purpose both temperature and concentration are taken as variable quantities at the cylindrical surface and away from it. The flow field situation is translated in terms of boundary value problem and the numerical simulation is carried out for their solution. Further, the effect logs of an involved physical flow parameters are explored through graphs and tables.

## Mathematical formulation

We have considered steady an incompressible and two dimensional mixed convection boundary layer stagnation point flow of non-Newtonian fluid model (Williamson fluid) brought by an inclined stretching cylinder. Flow situation is accounted with temperature and concentration stratification. The temperature as well as concentration at the surface of cylinder are assumed be higher in strength as compared to ambient temperature and concentration respectively. The boundary layer approximation reduces the flow controlling differential equations to forms given as follow:

(1) $$\frac{\partial \left(ru\right)}{\partial x}+\frac{\partial \left(rv\right)}{\partial r}=0\text{,}$$

(2) $$\begin{array}{l} u\frac{\partial u}{\partial x}+v\frac{\partial u}{\partial r}\;=\nu \left(\frac{1}{r}\frac{\partial u}{\partial r}+\frac{\partial^{2}u}{\partial r^{2}}+\frac{\Gamma }{\sqrt{2}r}{\left(\frac{\partial u}{\partial r}\right)}^{2}+\sqrt{2}\Gamma \frac{\partial u}{\partial r}\frac{\partial^{2}u}{\partial r^{2}}\right)+u_{s}\frac{\partial u_{s}}{\partial x}\\ +g\beta_{T}\left(T-T_{\infty }\right)+g\beta_{c}\left(C-C_{\infty }\right)\cos\alpha , \end{array}$$

(3) $$u\frac{\partial T}{\partial x}+v\frac{\partial T}{\partial r}=\frac{\kappa }{\rho {\text{c}}_{\text{p}}}\frac{\partial }{r\;\partial r}\left(\frac{\partial^{2}T}{\partial r^{2}}+\frac{1}{r}\frac{\partial T}{\partial r}\right)\text{,}$$

(4) $$u\frac{\partial C}{\partial x}+v\frac{\partial C}{\partial r}=D\left(\frac{\partial^{2}C}{\partial r^{2}}+\frac{1}{r}\frac{\partial C}{\partial r}\right)\text{,}$$

the axial axis of cylinder is supposed as *x*-axis and *r*-axis is perpendicular to it. The velocity components *u* is in the *x* direction while *v* in the *r* direction. Whereas, *ν*, *ρ*, *g*, *σ*, *β_T_*, *β_C_*, α denotes kinematic viscosity, fluid density, gravity, electrical conductivity, coefficient of thermal expansion, coefficient of concentration expansion and an inclination of cylinder with *x*-axis respectively. Whereas, *κ*, *c_p_*, and D, denotes thermal conductivity, specific heat at constant pressure and mass diffusivity respectively. The corresponding boundary conditions of problem are given by:

(5) $$u\left(x,\;r\right)=U(x)=ax,\;v(x,\;r)=0,\;T\left(x,\;r\right)=T_{w}(x)=T_{0}+\frac{bx}{L},\;C\left(x,\;r\right)=C_{w}(x)=C_{0}+\frac{dx}{L}\text{at}r=R\text{, and}\;u\left(x,\;r\right)\to u_{s}=a^{*}x,T\left(x,\;r\right)\to T_{\infty }(x)=T_{0}+\frac{cx}{L},\;C\left(x,\;r\right)\to C_{\infty }(x)=C_{0}+\frac{ex}{L}\text{as}r\to \infty \text{,}$$

where, *T_w_*(*x*), *C_w_*(*x*), *T_∞_*(*x*), *C_∞_*(*x*), *T*_0_, *C*_0_ denotes prescribed surface temperature, surface concentration, variable ambient temperature, variable ambient concentration, reference temperature and reference concentration respectively, where *b, c, d* and *e* are positive constants. Where, *ψ* is the stream function, which identically satisfies the continuity Eq. (1) and is defined as:

(6) $$u=\frac{1}{r}\left(\frac{\partial \psi }{\partial r}\right),\;v=\frac{-1}{r}\left(\frac{\partial \psi }{\partial x}\right)\text{.}$$

To trace out the solution of Eqs. (2)–(4) under boundary conditions given by Eq. (5), we used following transformation [24]:

(7) $$\begin{array}{l} u=\frac{U_{0}x}{L}f'(\eta ),\;\;v=-\frac{R}{r}\sqrt{\frac{U_{0}\nu }{L}}f\left(\eta \right),\;\;\eta =\frac{r^{2}-R^{2}}{2R}{\left(\frac{U_{0}}{\nu L}\right)}^{\frac{1}{2}},\;\\ \psi ={\left(\frac{U_{0}\nu x^{2}}{L}\right)}^{\frac{1}{2}}R\text{ f}(\eta ), \end{array}$$

where, *U*_0_ is the free stream velocity, *L* is the reference length, *f*(*η*) represents dimensionless variable so that, *f*' (*η*) is the velocity of fluid over an inclined stretching cylinder having radius *R*. In the field of fluid mechanics for particular problem a distinct set of similarity transformation may exist and can be obtained by using Lie group analysis. In addition, the boundary layer problems inherit some type of scaling symmetry due to the Buckingham Pi theorem [25], [26].

Once incorporating Eqs. (6)–(7) into Eqs. (2)–(4), we get

(8) $$\begin{array}{l} (1+2K\eta )f'''(\eta )+f(\eta )f''(\eta )-{\left(f'(\eta )\right)}^{2}+2Kf''(\eta )+\frac{3}{2}{\left(1+2K\eta \right)}^{\frac{1}{2}}K\lambda \;{\left(f''(\eta )\right)}^{2}\\ +\lambda \;{\left(1+2K\eta \right)}^{\frac{3}{2}}f''(\eta )f'''(\eta )+A^{2}+\lambda_{m}(\theta (\eta )+N\varphi (\eta ))\cos\alpha =0, \end{array}$$

(9) $$\left(1+2K\eta \right)\theta ''(\eta )+2K\theta '(\eta )+\mathrm{Pr}(f(\eta )\theta '(\eta )-f'(\eta )\theta (\eta )-f'(\eta )\beta_{1})=0\text{,}$$

(10) $$\left(1+2K\eta \right)\;\varphi ''(\eta )+2K\varphi '(\eta )+Sc\left(f(\eta )\varphi '(\eta )-f'(\eta )\varphi (\eta )-f'(\eta )\beta_{2}\right)=0\text{,}$$

the reduced boundary conditions are:

(11) $$\begin{array}{l} f(\eta )=0,\;\;f'\;(\eta )=1,\;\theta (\eta )=1-\beta_{1}\varphi (\eta )=1-\beta_{2},\;\text{at}\eta =0,\\ f'\;(\eta )\to \;\;A,\;\;\theta (\eta )\to 0\varphi (\eta )\to 0,\;\text{as}\eta \to \infty , \end{array}$$

where, *K*, *λ*, *λ_m_*, *N*, α, Pr, *β*_1_, *β*_2_, *A* and *Sc* denotes curvature parameter, Weissenberg number, mixed convection parameter, ratio of concentration to thermal buoyancy forces, Prandtl number, thermal stratification parameter, solutal stratification parameter, velocities ratio parameter and Schmidt number respectively and given as follow:

(12) K=1Rνa,    λ=Γ2U(x)3νx, λm=GrRex2,    N=Gr*Gr  anda=U0L,⁡Pr=μcpκ,    A=a*a, β1=cb,    Sc=νD,    β2=ed ,

where, *Gr* and Gr* denotes Grashof number due to temperature and concentration respectively and defined as:

(13) $$Gr=\frac{g\beta_{T}(T_{w}-T_{0})x^{3}}{\nu^{2}},\;\;\;Gr^{*}=\frac{g\beta_{C}(C_{w}-C_{0})x^{3}}{\nu^{2}}\text{.}$$

The skin friction coefficient is considered at the surface of cylinder and given as:

(14) $$C_{f}=\frac{\tau_{w}}{\rho \frac{U^{2}}{2}}\text{,}$$

(15) $$\tau_{w}=\mu {\left[\frac{\partial u}{\partial r}+\frac{\Gamma }{\sqrt{2}}{\left(\frac{\partial u}{\partial r}\right)}^{2}\right]}_{r=R}\text{,}$$

where, *μ* denotes viscosity of fluid and *τ_w_* is the shear stress. The dimensionless form of skin friction coefficient is given by

(16) $$\frac{1}{2}C_{f}Re_{x}^{1/2}=f^{\prime \prime }\left(0\right)+\frac{\lambda }{2}{\left(f^{\prime \prime }\left(0\right)\right)}^{2}\text{,}$$

with Rex=U0x2νL as a local Reynolds number.

The local Nusselt and Sherwood numbers are defined as:

(17) $$\begin{array}{l} Nu_{x}=\frac{xq_{w}}{k(T_{w}-T_{0})},\;\;q_{w}=-k{\left(\frac{\partial T}{\partial r}\right)}_{r=R}\text{,}\\ Sh=\frac{xj_{w}}{D(C_{w}-C_{0})},\;\;j_{w}=-D{\left(\frac{\partial C}{\partial r}\right)}_{r=R}, \end{array}$$

in dimensionless form, these quantities can be defined as:

(18) $$\begin{array}{l} Nu_{x}Re_{x}^{-1/2}=-\theta '\left(0\right),\\ ShRe_{x}^{-1/2}=-\varphi '\left(0\right). \end{array}$$

## Computational algorithm

The Eqs. (8)–(10) subjected to boundary conditions given by Eq. (11) is solved by employing shooting method with the aid of fifth order Runge-Kutta scheme. Firstly, reduction has been done in a system of seven first order simultaneous equations by letting

$$\begin{array}{l} M_{2}=f',\\ M_{3}=M{'}_{3}=f'',\\ M_{5}=\theta ',\\ M_{7}=\varphi ', \end{array}$$

then the equivalent form of Eqs. (8)–(10) under new variables is given by:

(19) $$\left[\begin{array}{l} M{'}_{1}=M_{2}\\ M{'}_{2}=M_{3}\\ M{'}_{3}=\frac{{\left(M_{2}\right)}^{2}-M_{1}M_{3}-(2K)M_{3}-\frac{3}{2}\lambda K{\left(1+2K\eta \right)}^{\frac{1}{2}}{M_{3}}^{2}-\lambda {\left(1+2K\eta \right)}^{\frac{3}{2}}M_{3}M_{4}-A^{2}-\lambda_{m}(M_{4}+NM_{6})\cos\alpha }{(1+2K\eta )+\lambda {\left(1+2K\eta \right)}^{\frac{1}{2}}M_{3}}\\ M{'}_{4}=M_{5}\\ M{'}_{5}=\frac{\mathrm{Pr}(M_{2}M_{4}+\beta_{1}M_{2}-M_{1}M_{5})-2KM_{5}}{1+2K\eta }\\ M{'}_{6}=M_{7}\\ M{'}_{7}=\frac{Sc(M_{2}M_{6}+\beta_{2}M_{2}-M_{1}M_{7})-2KM_{7}}{1+2K\eta } \end{array}\right]$$

the corresponding boundary conditions in new variables are given as follows:

(20) $$\begin{array}{l} M_{1}\left(0\right)=0,\\ M_{2}\left(0\right)=1,\\ M_{3}\left(0\right)=unknown,\\ M_{4}\left(0\right)=1-\beta_{1},\\ M_{5}\left(0\right)=unknown,\\ M_{6}\left(0\right)=1-\beta_{2},\\ M_{7}\left(0\right)=unknown \end{array}$$

In order to integrate Eq. (19) as an initial value problem, we required values for $M_{3}\left(0\right)$i.e.F''(0), $M_{5}\left(0\right)$ i.e. θ'(0) and $M_{7}\left(0\right)$ impliesφ'(0). The initial conditions$M_{3}\left(0\right)$,$M_{5}\left(0\right)$,$M_{7}\left(0\right)$ are not given but we have additional boundary conditions:

(21) $$\begin{array}{l} M_{2}\left(\infty \right)=0,\\ M_{4}\left(\infty \right)=0,\\ M_{6}\left(\infty \right)=0. \end{array}$$

By choosing favourable guessed values of f''(0), θ'(0)and φ'(0), the integration of system of first order differential equations are carried out in a such a way that the boundary conditions given in Eq. (21) holds absolutely. The step size Δη = 0.05 is used to obtain the numerical solution with four decimal accuracy as convergence criteria.

## Results and discussion

**Fig. 1:**
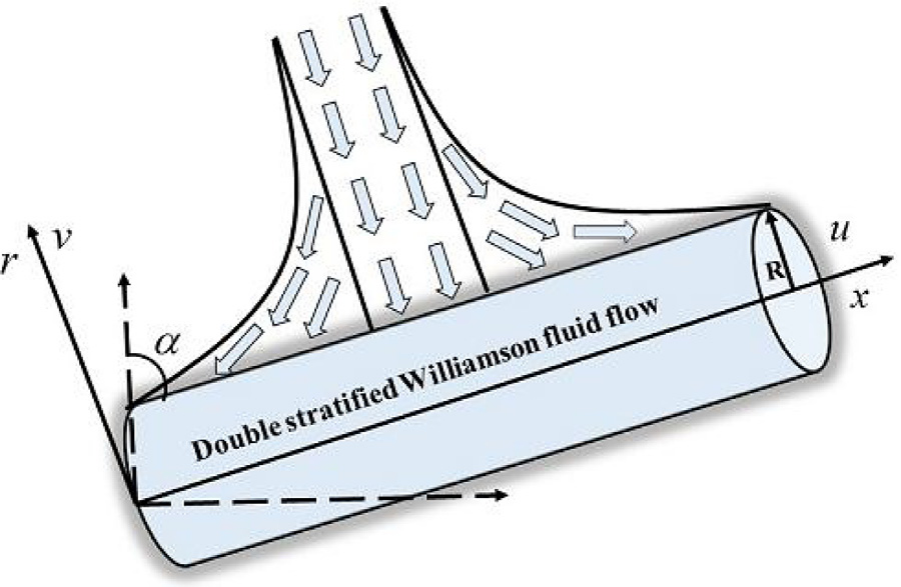
Schematic diagram of problem.

**Fig. 2:**
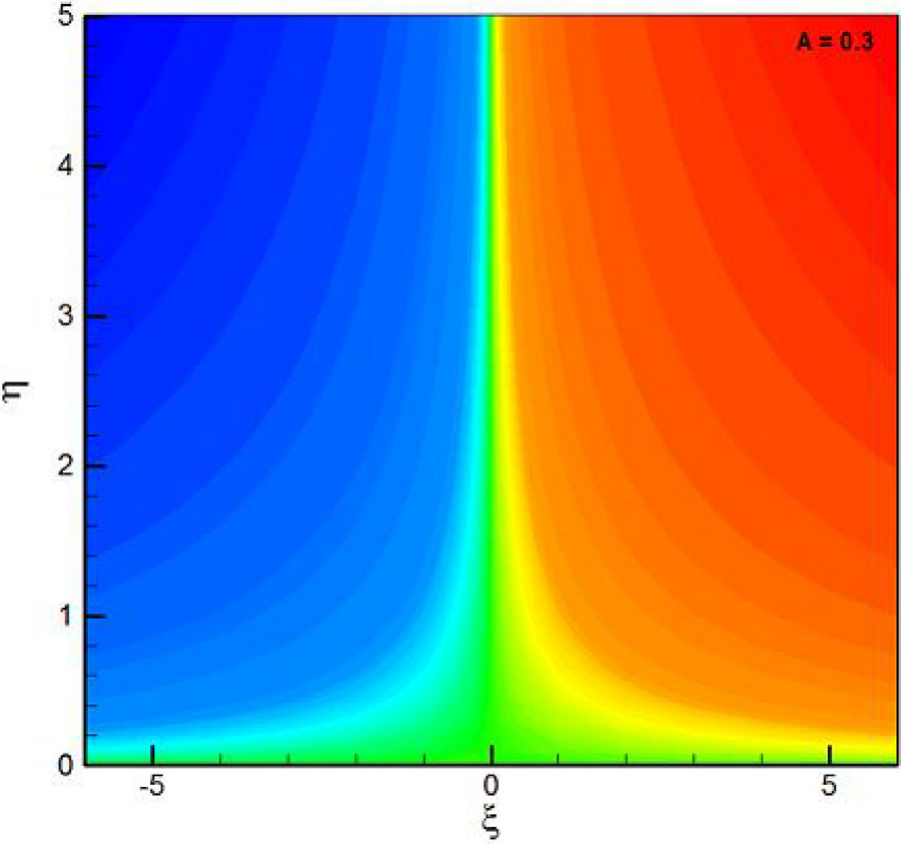
Stream lines pattern for *A* < 1.

**Fig. 3:**
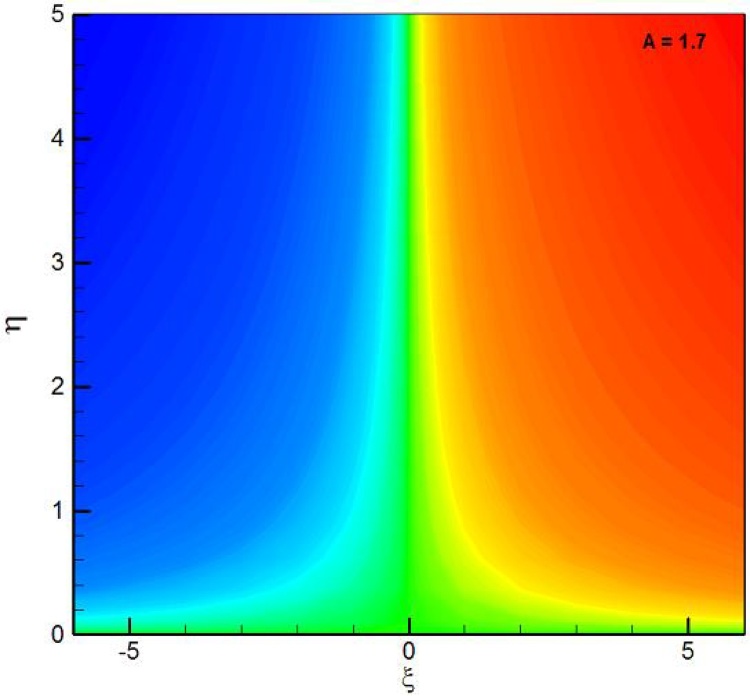
Stream lines pattern for *A* > 1.

Table 1, Table 2, Table 3, Table 4 are used to examine the variations of physical quantities namely, skin friction coefficient, heat transfer rate and mass transfer rate. Particularly, Table 1, Table 2 provides the alterations of skin friction coefficient for positive values of thermal stratification parameter, solutal stratification parameter, curvature parameter, Weissenberg number, Prandtl number and Schmidt number. It is found from Table 1, Table 2 that the skin friction coefficient shows higher values for thermal stratification parameter, solutal stratification parameter, curvature parameter, Prandtl number and Schmidt number while it shows decline nature towards Weissenberg number. The negative sign of skin friction coefficient physically shows that the cylindrical surface exerts drag force on fluid particles. Table 3 presented numerical values (in absolute sense) for heat transfer rate towards higher values of thermal stratification parameter, curvature parameter, and Prandtl number. It is observed that the heat transfer rate shows an inciting values for thermal stratification parameter, curvature parameter, and Prandtl number. The negative values of Nusselt number shows transfer of heat normal to the cylindrical surface. Whereas, Table 4 is used to identified the influence of curvature parameter, Schmidt number and solutal stratification parameter on mass transfer rate. It is observed that mass transfer rate shows an inciting nature for lager values of curvature parameter, Schmidt number and solutal stratification parameter. The negative values of Sherwood number shows mass transfer normal to the cylindrical surface. The Eqs. (2)–(4) along with Eq. (5) narrates heat and mass transfer characteristics of mixed convection Williamson stagnation point flow due to an inclined stretching cylindrical surface. In the absence of heat and mass transfer characteristics if we used K = λ = λ_m_ = 0, the current reduced to study reported by both [22] and [23]. Therefore, Table 5 is constructed to offered comparison of skin friction coefficient towards different values of velocities ratio parameter. An excellent match is found which confirms the present attempt. Fig. 1 depicts the geometry of flow model. Fig. 2 is plotted to examine the stream lines pattern for *A* < 1, in other words when stretching velocity exceeds against free stream velocity. Physically, the boundary layer thickness increases largely when stretching velocity is dominant as compared to free stream velocity. Further, the involvement of free stream velocity is little significant but lesser then stretching velocity so that the particle disturbance is significant at cylindrical surface as compared to far away from surface. In Fig. 3 stream lines are switches accordingly to the velocities ratio parameter *A* > 1. In this case free stream velocity exceeds stretching velocity. The thickness of boundary layer decreases when *A* increases. In actual when *A* increases, we yields increase in free stream velocity for fixed values of stretching velocity, ultimately straining motion adjacent to the stagnation region brings an inciting acceleration of free stream as a result thinning of boundary layer is observed.

**Table 1:** Numerical values of skin friction coefficient for λ, Sc, β_1_ and β_2_.

λ	Sc	$\beta_{1}$	$\beta_{2}$	f''(0)	12CfRex12=f''(0)+λ2(f''(0))2
0.1	0.1	0.1	0.1	−1.2731	−1.3930
0.2	–	–	–	−1.2944	−1.0902
0.3	–	–	–	−1.3312	−1.0065
–	0.1	–	–	−1.2731	−1.3930
–	0.2	–	–	−1.2734	−1.3934
–	0.3	–	–	−1.2741	−1.3939
–	–	0.1	–	−1.2731	−1.3930
–	–	0.2	–	−1.2743	−1.3954
–	–	0.3	–	−1.2754	−1.3968
–	–	–	0.2	−1.2766	−1.3951
–	–	–	0.3	−1.2785	−1.3963

**Table 2:** Skin friction coefficient friction for various values of *K*, and *Pr*.

K	Pr	f''(0)	12CfRex12=f''(0)+λ2(f''(0))2
0.1	0.1	−0.1935	−0.3133
0.2	–	−0.2539	−0.3341
0.3	–	−0.3041	−0.3475
–	0.1	−0.1935	−0.3133
–	0.2	−0.1939	−0.3145
–	0.3	−0.1944	−0.3168

**Table 3:** Heat transfer rate at the outer surface of cylinder for varies values of K, Pr and β_1_.

*K*	Pr	$\beta_{1}$	θ '(0)
0.1	0.1	0.1	−0.7340
0.2	–	–	−0.7837
0.1	–	–	−1.0002
–	0.1	–	−0.7340
–	0.2	–	−0.9874
–	0.3	–	−1.0132
–	–	0.1	−0.7340
–	–	0.2	−0.7835
–	–	0.3	−1.0031

**Table 4:** Mass transfer rate at outer surface of cylinder for different values of K, *Sc* and *β*_2_.

K	Sc	$\beta_{2}$	φ'(0)
0.1	0.1	0.1	−0.8283
0.2	–	–	−0.9479
0.3	–	–	−1.1243
–	0.1	–	−0.8283
–	0.2	–	−1.0012
–	0.3	–	−1.0654
–	–	0.1	−0.8283
–	–	0.2	−0.9935
–	–	0.3	−1.1538

**Table 5:** Comparison of skin friction coefficient for various values of *A*.

A	Mahapatra and Gupta [22]	Nazar et al. [23]	Present outcomes
0.10	−0.9694	−0.9694	−0.9694
0.20	−0.9181	−0.9181	−0.9182
0.50	−0.6673	−0.6673	−0.6675
2.00	2.0175	2.0176	2.0179
3.00	4.7293	4.7296	4.7298

**Fig. 4:**
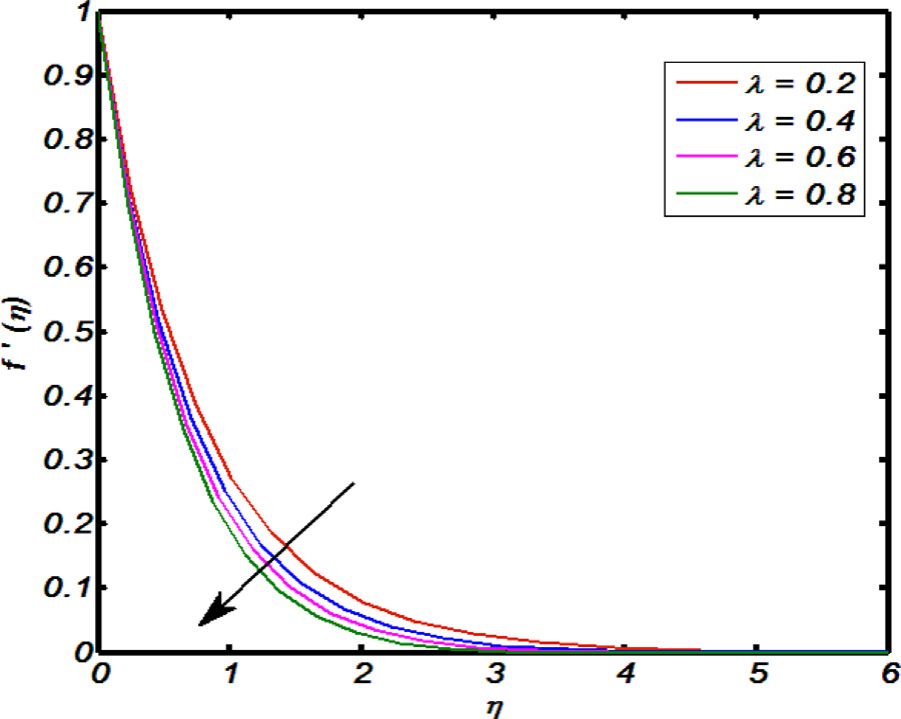
Effect of Weissenberg number over velocity profile.

**Fig. 5:**
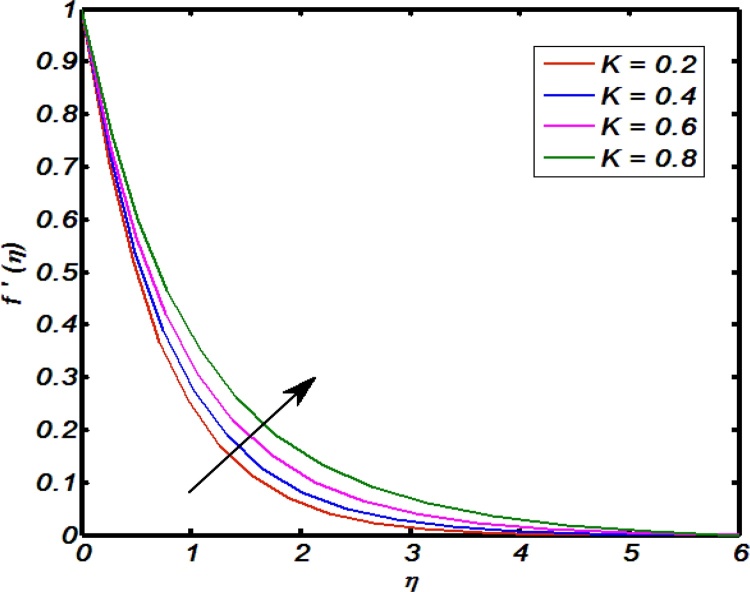
Effect of curvature parameter over velocity profile.

**Fig. 6:**
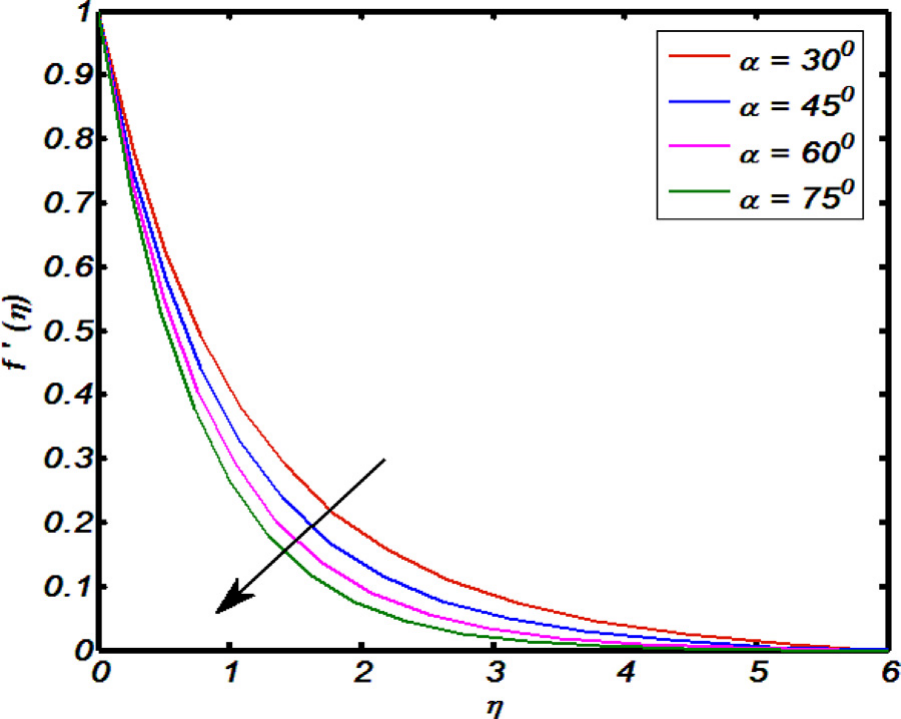
Effect of an inclination over velocity profile.

**Fig. 7:**
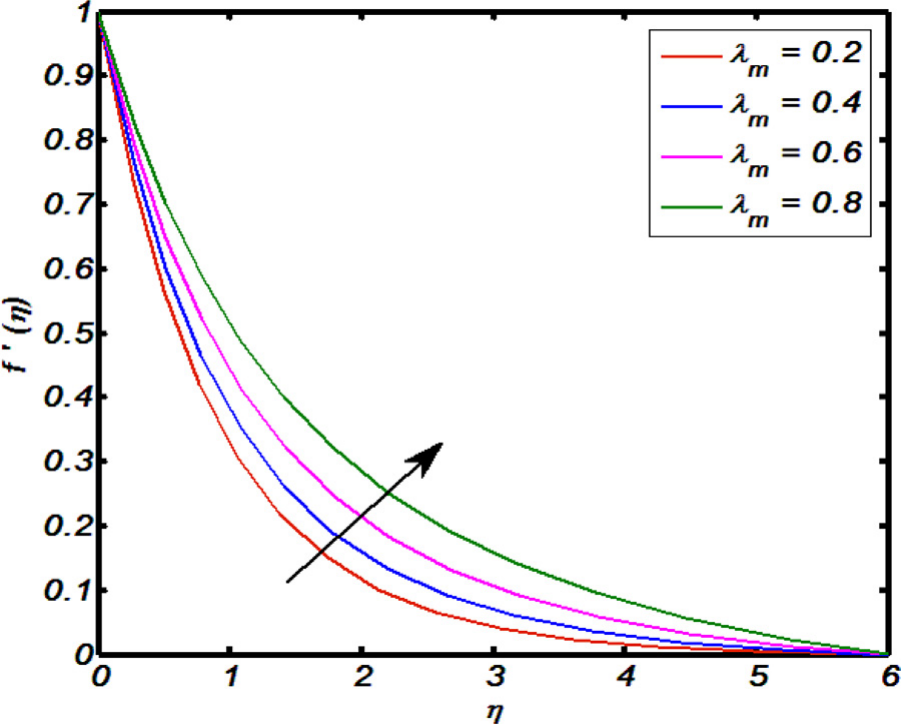
Effect of mixed convention parameter over velocity profile.

**Fig. 8:**
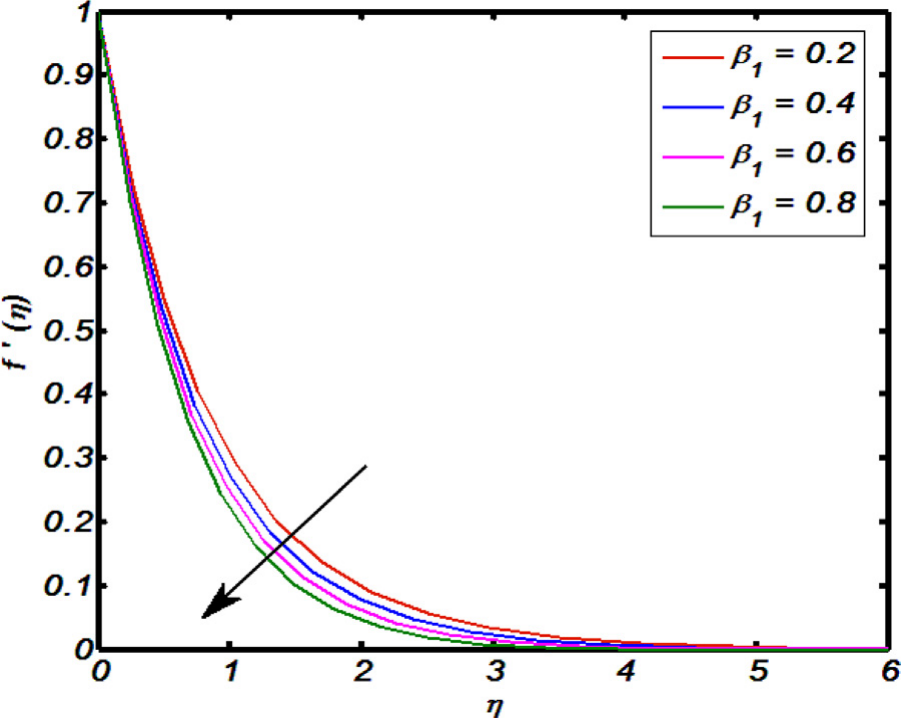
Effect of thermal stratification parameter over velocity profile.

Fig. 4, Fig. 5, Fig. 6, Fig. 7, Fig. 8 are used to identify that how velocity profile are effected by the controlling parameters namely, thermal stratification parameter, mixed convection parameter, an inclination, curvature parameter and Weissenberg number. Fig. 4 paints the impact of Weissenberg number on velocity profile. It is seen that an increase in Weissenberg number brings decline values in velocity profile. The fact behind is that on increasing values of Weissenberg number the relaxation time of the fluid increases which creates resistance to the fluid particles so that the velocity of the fluid decreases. Fig. 5 indicates that for high values of curvature parameter the velocity profile increases. The reason is that for higher values of curvature parameter the radius of cylinder decreases. Hence less will be the contact surface area which produces less amount of resistance towards fluid particles so that the velocity profile shows an inciting values. Fig. 6 tells that there is an inverse relation between an inclination and velocity profile. For large values of an inclination the velocity profile decreases. The fact behind is that an increase in inclination about *x*-axis the effect of gravity reduces as a result velocity profile decreases with in boundary layer. The effect of mixed convention parameter over velocity profile is shown in Fig. 7. For higher values of mixed convention parameter the velocity profile increases with in a boundary layer. Physically, this is because of the enhancement in buoyancy force. Fig. 8 identify that there is inverse relation between thermal stratification parameter and velocity distribution that is an increase in thermal stratification parameter the velocity profile decreases. This effect is due to drop of convective potential between surface of cylinder and ambient temperature.

**Fig. 9:**
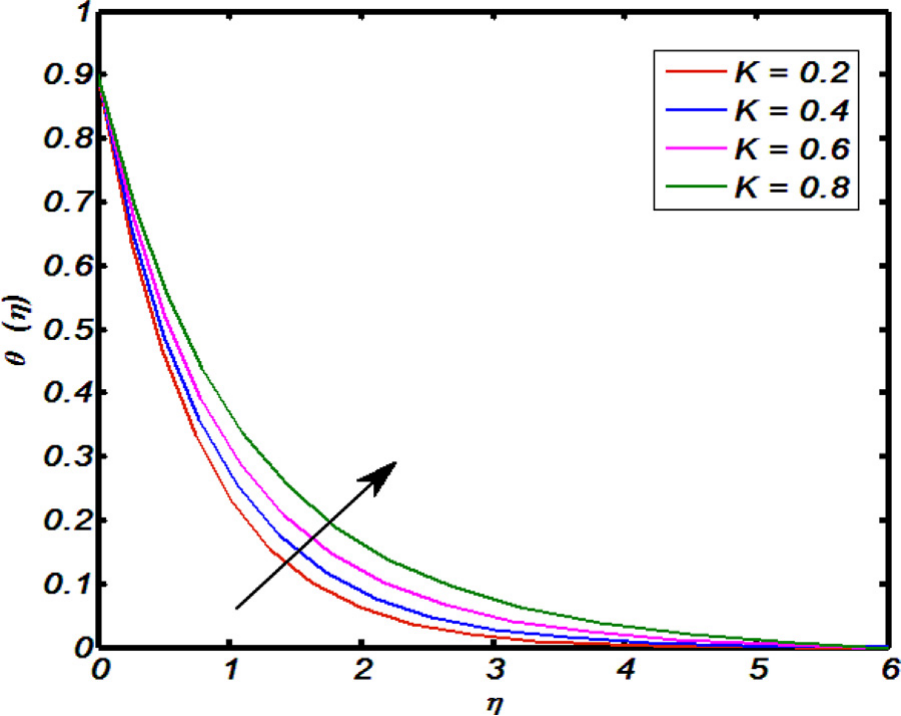
Effect of curvature parameter over temperature profile.

**Fig. 10:**
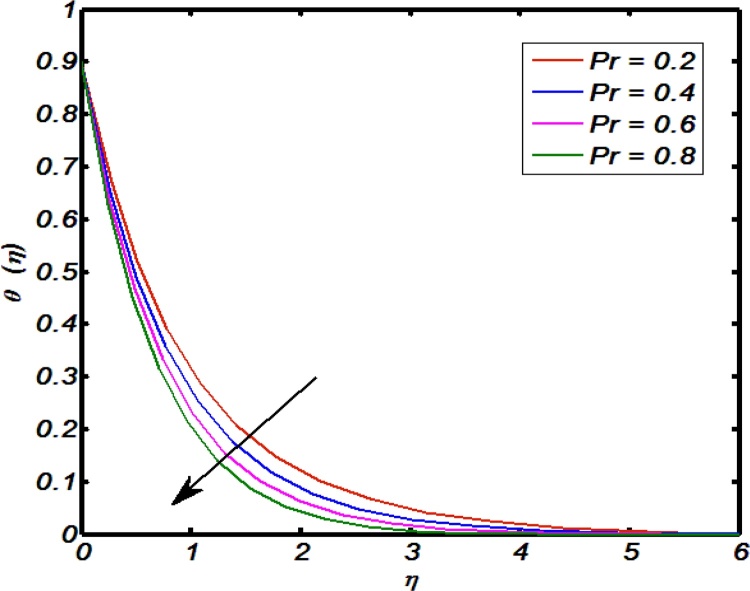
Effect of Prandtl number over temperature profile.

**Fig. 11:**
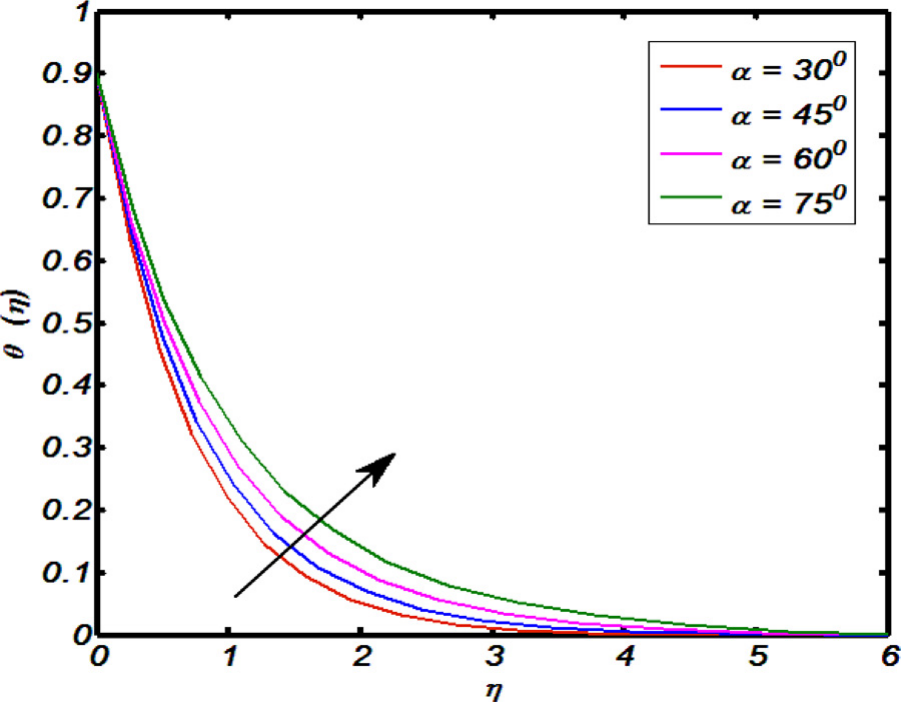
Effect of an inclination over temperature profile.

**Fig. 12:**
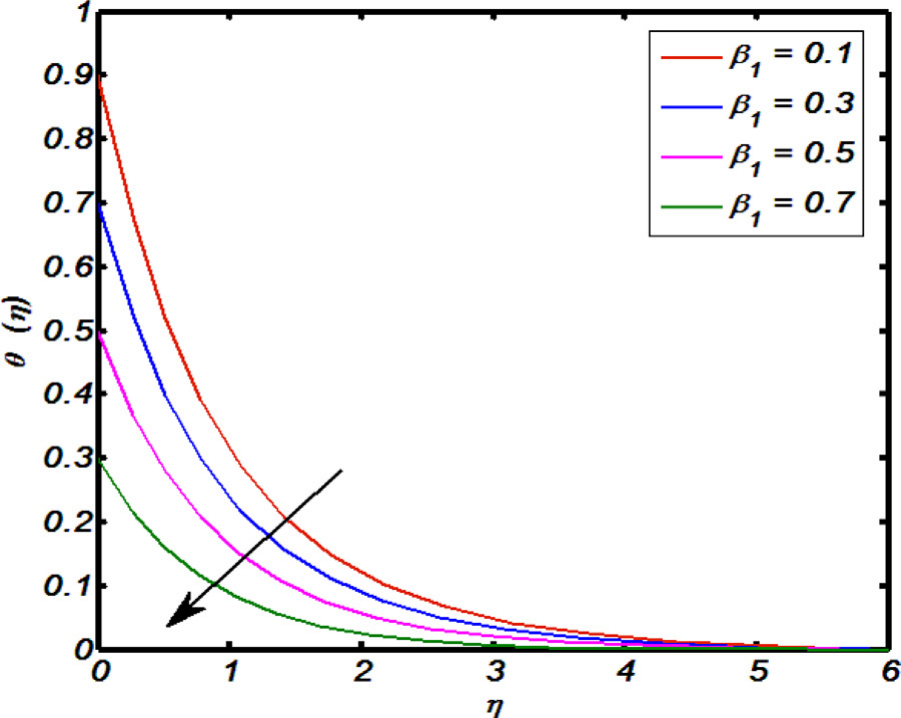
Effect of thermal stratification parameter over temperature profile.

**Fig. 13:**
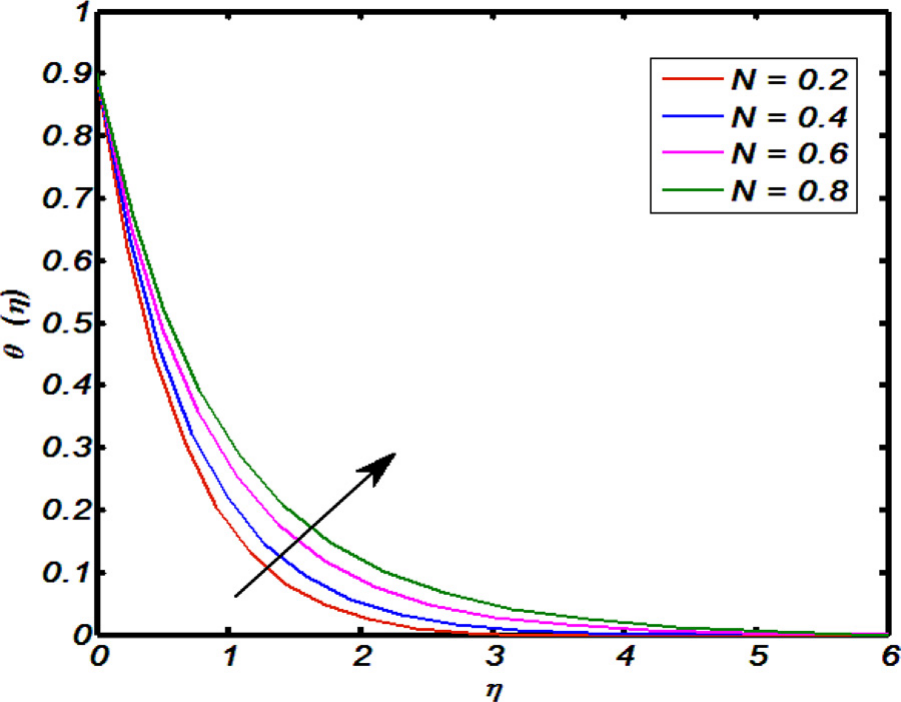
Effect of ratio of buoyancy forces over temperature parameter profile.

**Fig. 14:**
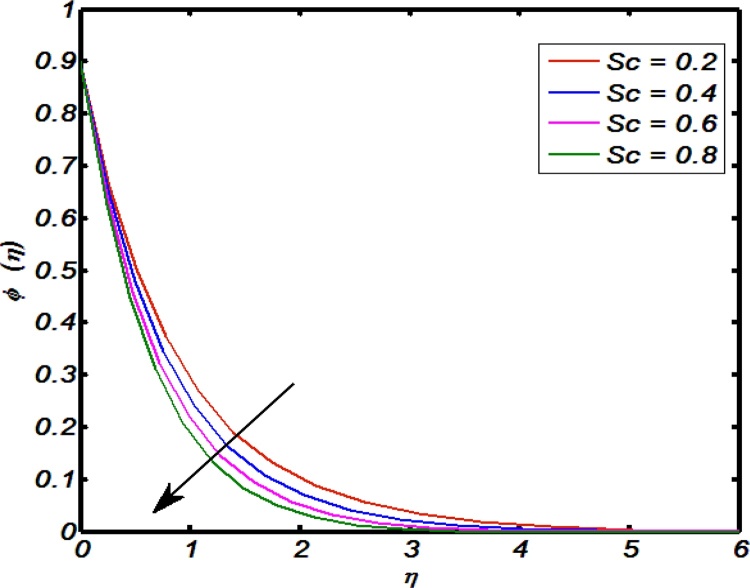
Effect of Schmidt number on concentration profile.

**Fig. 15:**
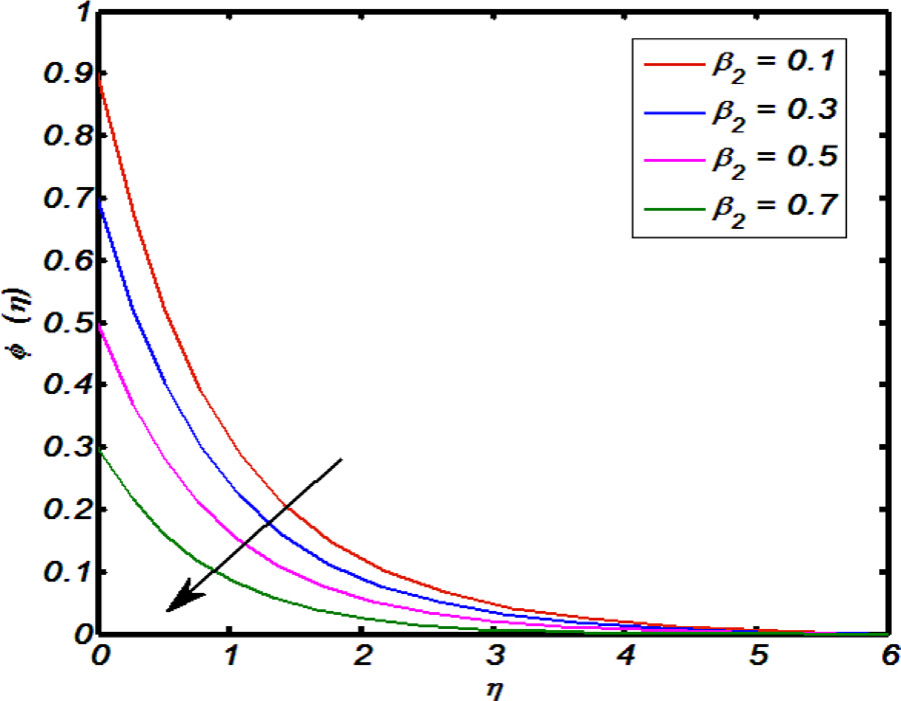
Effect of solutal stratification parameter over concentration profile.

Fig. 9, Fig. 10, Fig. 11, Fig. 12, Fig. 13 shows that the effects of flow controlling parameters namely, an inclination, Prandtl number, curvature parameter, thermal stratification parameter, ratio of buoyancy forces over temperature profile. Particularly, Fig. 9 indicates that for higher values of curvature parameter the temperature distribution increases. In actual, Kelvin temperature is an average kinetic energy so, an increase in curvature parameter cause decrease in radius of curvature due to which velocity of the fluid particles enhances ultimately, average kinetic energy increase which yields an increment in temperature profile. Fig. 10 depicts that there is an inverse relation between Prandtl number and fluid temperature. The higher values of Prandtl number reflects weak energy diffusion. So, an increase in Prandtl number results a strong reduction in temperature profile of the fluid which causes thinner boundary layer. Fig. 11 identify that there is an inverse relation between an inclination and temperature profile. For large values of inclination the temperature profile decreases. The fact behind it is that an increase in an inclination about *x*-axis the effect of gravity reduces which brings decline in temperature profile. Fig. 12 identify that there is an inverse relation between thermal stratification parameter and temperature distribution that is that an increase in thermal stratification parameter the temperature profile decreases. This effect is because of drop of convective potential between surface of cylinder and ambient once. Fig. 13 shows the impact of ratio of concentration to the thermal buoyancy forces on temperature distributions. For large values of thermal buoyancy forces the temperature profile increases. Fig. 14, Fig. 15 illustrates that how concentration profile are effected by Schmidt number and solutal stratification parameter. In detail, Fig. 14 shows that for higher values of Schmidt number the concentration profile decreases. Because Schmidt number has an inverse relation with mass diffusivity so larger values of Schmidt number gives thinning in the concentration boundary layer as a result concentration profile decreases. Fig. 15 identify that an increase in solutal stratification parameter the concentration distribution decreases. This effect is similar with the relation of thermal stratification parameter verse temperature profile.

## Conclusion

A double stratified mixed convection stagnation point boundary layer flow of Williamson fluid flow is considered towards an inclined stretching cylinder. The flow field is translated in terms of partial differential equations. These partial differential equations are converted into ordinary differential equations. A numerical algorithm is executed to obtain concerned results and the effects of pertinent flow controlling parameters on dimensionless velocity, temperature and concentration are offered with the aid of graphs. The key results are listed as follow:

• The compatibility of endpoint conditions is validated by providing stream lines pattern (see Fig. 2 and Fig. 3) towards velocities ratio parameter.
• The fluid velocity shows an inciting nature for positive value of mixed convection parameter and curvature parameter but it shows opposite trends for the thermal stratification parameter, an inclination and Weissenberg number.
• The fluid temperature reflects higher attitude towards curvature parameter, an inclination and ratio of buoyancy forces but an inverse attribute is found for both thermal stratification parameter and Prandtl number.
• The fluid concentration profile decreases for higher values of both solutal stratification parameter and Schmidt number.
• It is noticed that the skin friction coefficient shows an inciting nature for thermal stratification parameter, solutal stratification parameter, curvature parameter, Prandtl number and Schmidt number but it shows decline nature towards Weissenberg number.
• The heat transfer rate (normal to the cylindrical surface) increases for higher values of curvature parameter, Prandtl number and thermal stratification parameter.
• The mass transfer rate (normal to the cylindrical surface) shows an inciting behaviour for curvature parameter, Schmidt number and solutal stratification parameter.
